# Translating Lupus: Comparative Transcriptional Profiles of Preclinical Lupus Models and Their Relevance to Human Disease

**DOI:** 10.3390/biology13100778

**Published:** 2024-09-28

**Authors:** James T. Parker, Ching-Yun Chang, Kara Kersjes, Ixavier A. Higgins, Andrew C. Vendel, William Y. Chang

**Affiliations:** 1 Immunology Discovery Research, Lilly Biotechnology Center, Eli Lilly and Company, San Diego, CA 92121, USA; parker_james@lilly.com (J.T.P.); kersjes_kara@lilly.com (K.K.); chang_william_y@lilly.com (W.Y.C.); 2 Discovery Statistics, Eli Lilly and Company, Indianapolis, IN 46225, USA; veavi.chang@lilly.com (C.-Y.C.); higgins_ixavier@lilly.com (I.A.H.)

**Keywords:** lupus, mouse models, differentially expressed genes, scRNA-seq, mouse-to-human translation

## Abstract

**Simple Summary:**

Mouse models of disease are commonly used to generate compelling data which are the basis for biological conviction in drug discovery. Systemic lupus erythematosus (SLE) is a debilitating autoimmune disease which can often lead to kidney failure, and there are many mouse models that claim to mimic the disease, but none can completely reproduce all aspects of human SLE. To effectively utilize these models for drug development, we must understand which biological similarities they share with human SLE patients. Here, we compared a select number of lupus mouse models to see how well immune pathways in kidney samples mimic human disease in kidney samples from lupus patients. The mouse models vary in the type and intensity of immune responses that were observed. Each mouse model shares various aspects of immune response with human SLE patients and should be utilized based on drug targets and shared biological pathways.

**Abstract:**

Systemic lupus erythematosus (SLE) is a chronic, systemic autoimmune disease which can present with mixed organ involvement. Kidney involvement in lupus nephritis (LN) is a severe complication and major cause of mortality in SLE patients, second only to cardiovascular disease. While mouse models have helped uncover some molecular pathways involved in SLE/LN, we need a better understanding of the connection of these pathways and the immune cells involved in disease pathogenesis to develop new and effective therapies. Furthermore, models used for studying SLE/LN in mice have a heterogeneous immune response and may not always represent disease manifestations observed in patients. Identifying models that have shared pathways with human disease would allow for better translation for developing effective SLE/LN therapies. The molecular pathways of five different SLE/LN models (MRL/lpr, poly (I:C)-induced, interferon-α-induced, bm12 GvHD, and spontaneous NZB/W F1) were compared to characterize the immune response in mouse kidneys. These models demonstrated varied magnitudes in immune responses and proportions of innate vs. adaptive cell involvement. These findings were compared to human molecular pathways and cell types from public databases, including the Accelerating Medicine Partnership–Systemic Lupus Erythematosus Program (AMP-SLE), to help corelate mechanisms involved in mouse models to human disease.

## 1. Introduction

SLE is a chronic debilitating disease that can involve multiple organs including kidney, skin, lung, skeletal muscle, joints and the central nervous system [1,2]. The pathogenesis of SLE is complex and is characterized by a generation of autoantibodies against double-stranded DNA (dsDNA), deposition of immune complexes, and recruitment of immune cells (both adaptive and innate). Lupus nephritis (LN) occurs in approximately half of the SLE patients and can result in life-threatening end-stage renal disease in 10% of the LN patients [3]. Human LN is heterogeneous as exemplified by the six classes of LN defined by the International Society of Nephrology and Renal Pathology Society Classification (ISN/RPS) [4]. The classification distinguishes the classes by varying manifestations of glomerular, vascular, and tubular pathologies with differing degrees of inflammation observable under light microscopy [4]. Immunophenotyping of the inflammatory response in LN was more recently detailed in the single-cell analysis of renal samples from the ambitious AMP program. The program identified a mixture of immune cells that contained myeloid cells, B cells, T cells, and NK cells among the inflammatory population in kidney samples from human LN patients [5].

Mouse models of LN have been instrumental in elucidating the pathogenesis of SLE. One of the first rodent lupus models was described in 1966 after crossing New Zealand black mice (NZB) to New Zealand white mice (NZW) [6]. The NZB/W mice became moribund typically between 7 and 13 months of age and developed LN spontaneously [6]. This classical model of lupus nephritis is responsive to treatments targeting IL-6 [7], Bruton’s tyrosine kinase (BTK) inhibitor [8,9], CD20 [10], BAFF [11], glucocorticoid receptor [12], and CTLA-4 [13]. The variability observed with onset of disease and rate of progression along with the delayed onset of disease makes this model inefficient and not amenable to drug discovery research. To accelerate and synchronize the onset of disease in the NZB/W mice, strategies to activate immune cells using interferon-a (IFNa) [14,15] and polyinosinic/polyctidylic acid (poly (I:C)) [16,17] have been used. These strategies not only accelerated the NZB/W model, but also had distinct effects on the immune response. For example, in the IFNa-induced model, the mice were resistant to CTLA-4 treatment and inflammation was less apparent in the early stages of disease [15,18]. Alternatively, the poly (I:C)-induced model enhanced the recruitment of macrophages to the kidney [16].

Another widely characterized and utilized LN mouse model is the MRL/lpr model. These mice have a recessive lymphoproliferation (lpr) mutation in Fas that leads to a loss of function and failure of the lymphocytes to undergo programmed cell death [19]. The uncontrolled proliferation of lymphocytes results in lymphadenopathy, splenomegaly, and anti-nuclear antibodies (ANAs) [20]. Immune complex deposition in the kidneys of MRL/lpr mice leads to glomerulonephritis characterized by an infiltration of neutrophils and macrophages along with lymphocytes [21,22]. Another LN mouse model is the inducible chronic graft-versus-host disease (GvHD) model which utilizes the B6(C)-*H2-Ab1^bm12^*/KhEgJ (bm12) mice. The bm12 are identical to C57BL/6 (B6) mice except for three amino acid differences in the MHC class II. Transferring splenocytes from bm12 mice to B6 mice (or vice versa) will lead to an expansion of T follicular helper cells (Tfh), germinal center (GC) B cells, and the development of ANAs [23]. The model is characterized by mild glomerulonephritis and is associated with the activation of alloreactive donor T cells to recipient antigen-presenting cells (APCs), leading to IgG deposition in the kidneys [24].

To date, no study has directly compared the immune response in kidneys of the various LN mouse models. The goal of this paper was to immunophenotype the inflammatory response in the kidneys of the spontaneous NZB/W, IFNα-induced NZB/W, poly (I:C)-induced NZB/W, MRL/lpr, and bm12 mouse LN models. Since none of the LN mouse models completely recapitulate human LN, we were also interested in assessing how the models compare to human LN. To that end, we compared the various LN mouse renal immune responses to publicly available whole transcriptome RNA sequencing of renal tissue from human SLE/LN to demonstrate potential similarities.

## 2. Materials and Methods

### 2.1. Mouse Husbandry

Mice were housed in Innovive Mouse Caging System (Innocage^®^) in a 12 h light/dark cycle with the ambient temperature set at 70 °F. Cage bottoms were delivered double-bagged and irradiated for added biosecurity. Bags of caging (25 cages per bag) with pre-filled 1/8” Innocob bedding or ALPHA-dri^®^ bedding were stored in the animal facility storage room off the floor on metroplex-type racks at least 6 inches away from the wall. In addition, animal care technicians sprayed the outside of each bag with quatricidal solution before storing and conducting a visual inspection. Cage lids were low-profile ventilation-area static mouse lids with large dual Uniblend filter areas and metal grates. Rodents were provided with Teklad Global Protein diet (irradiated) ad libitum which comes in vacuum-sealed 33 lb bags. Water was provided ad libitum through 100% PET plastic mouse water bottles pre-filled with ultra-purified water. Mice were transferred to new cages in a cage-changing station following standard operating procedures. Monitoring for infectious agents was accomplished through the institutional Laboratory Animal Health Monitoring Program. Sentinel animals within each animal holding room, along with vendor controls, were screened for infectious agents on a quarterly basis. At the same time, rodents were tested for the presence of pinworms and hair/skin mites.

### 2.2. Accelerated Mouse Models of SLE

#### 2.2.1. Poly (I:C)-Induced Lupus Nephritis Model

Female NZB/W F1 mice (12 weeks old) were obtained from The Jackson Laboratory (Bar harbor, ME). To assess the change in urinary protein and circulating autoantibodies over time, baseline serum and urine were collected prior to poly (I:C) injections. High-molecular-weight poly (I:C) (1.5–8 kb) (InvivoGen, San Diego, CA, USA) was dissolved to 1 mg/mL concentration in physiological water (NaCl 0.9%) and administered intraperitoneally (i.p.) at a 2:1 volume of 200 µL/mouse, 3 times per week until day 28. Mice were euthanized 8 weeks post initial poly (I:C) injection and kidneys were collected for gene expression analysis.

#### 2.2.2. Mouse IFNα (mIFNα)-Induced Lupus Nephritis Model

Female NZB/W F1 mice (12-weeks old) were obtained from Jackson Laboratories (Bar Harbor, ME, USA). Mice were administered 10^11^ genome copies of LacZ adeno-associated virus 8 (AAV8) or mouse IFNα5 AAV8 (Vector Biolabs, Malvern, PA, USA) via intravenous injection (i.v.) in the lateral tail vein at a volume of 100 µL/mouse. Mice were euthanized and kidneys were collected from the mice 6 weeks post AAV injection for gene expression analysis.

#### 2.2.3. Bm12 Lupus Nephritis Model

Male B6(C)-*H2-Ab1^bm12^*/KhEgJ (bm12) and B6.SJL-*Ptprc^a^ Pepc^b^*/BoyJ (Pepboy) mice were obtained from Jackson Laboratories (Bar Harbor, ME, USA). Splenocytes (10^8^ cells per mouse) from 11-week-old bm12 mice were transferred i.p. into 11-week-old Pepboy recipient mice. Mice were euthanized and kidneys were collected from the mice 6 weeks after splenocyte transfer for gene expression analysis.

### 2.3. Spontaneous Mouse Models of SLE

#### 2.3.1. NZB/W Spontaneous Lupus Nephritis Model

The 16-week-old female NZB/W mice were obtained from Jackson Laboratories (Bar Harbor, ME, USA) and allowed to age in-house. Mice were euthanized at 42 weeks of age and kidneys were collected for gene expression analysis.

#### 2.3.2. MRL/Lpr Lupus Model

Eight-week-old female MRL/MpJ-*Fas^lpr^*/J (MRL/lpr) and female MRL/MpJ (MRL) mice were obtained from Jackson Laboratories (Bar Harbor, ME, USA). Mice were euthanized and kidneys were collected from the mice at 27 weeks of age for gene expression analysis.

### 2.4. Anti-Double-Stranded DNA (dsDNA) Assay

Serum anti-dsDNA levels were quantitated using the Meso Scale Discovery (MSD) electrochemiluminescence platform. A binding plate was blocked overnight at 4 °C with 100 µg/mL of calf thymus DNA in PBS. Following blocking, the plate was washed 3 times with PBS + 0.1% Tween20, and 25 µL of MSD diluent 41 was added per well. The plate was then incubated at room temperature (RT) overnight. After overnight incubation, the plate was washed again 3 times. Standards were prepared by making a 5-fold serial dilution of a high titer mouse serum stock in MSD diluent 41. Serum samples from the study were diluted 1:250 in diluent 41. Both diluted samples and standards were added to the plate (25 µL/well) and incubated at RT for 2 h. Post-incubation, the plate was washed 3 times, followed by the addition of 25 µL of 1x goat anti-mouse sulfo-Tag antibody and a 1 h incubation at RT. The plate was washed again and 150 µL of 2x Read Buffer T in DI water was added. Finally, the plate was read immediately on the MSD reader and analyzed using the MSD discovery workbench.

### 2.5. Quantification of RNA from Mouse Tissue

Mouse kidney tissues were disrupted using 2 mL lysing matrix S tubes (MP Bio, Irvine, CA, USA) and mini-beadbeater 96 homogenizer (BioSpec, Bartlesville, OK, USA). RNA was purified from mouse kidneys using the Qiagen miRNeasy 96 kit (QIAGEN, Venlo, NL, USA). For each mouse model, RNA was purified from at least 3 individual naive and diseased kidneys. A total of 100 ng of purified RNA from each kidney was quantified with the NanoString nCounter platform using the Mouse Autoimmune Profiling multiplex panel (NanoString, Seatle, WA, USA). The AdvancedAnalysis (Version 2.0) nCounter module was used to quality control and normalize the RNA samples. All samples passed quality control for positive spike-in RNA titration and normalization variance. Normalized counts were used in downstream analyses detailed in the statistical analysis methods. The amounts of naïve and diseased mice analyzed for each animal model are as follows: MRL/lpr (n = 5 naïve and 7 diseased), poly (I:C) (n = 3 naïve and 9 diseased), mIFNa (n = 5 naïve and 7 diseased), NZB/W (n = 6 naïve and 6 diseased), and GvHD (n = 3 naïve and 9 diseased).

### 2.6. Statistical Analysis

#### 2.6.1. Per-Marker Analysis in Animal Models

Per-marker analyses in animal models are applied with a one-way ANOVA model on log2 gene expression with comparison of interest between the diseased and naive group. Multiplicity adjustment across markers is applied with the Benjamini–Hochberg method. Fold change and adjusted *p*-value are reported for each marker. Differentially expressed genes (DEGs) are defined as an up- or down-regulated diseased group compared to a naïve group with an adjusted *p*-value threshold <0.1 and at least a 1.5-fold change.

#### 2.6.2. Pathway Analysis of Differential Expression in Animal Models

Pathway analysis in animal models is summarized as the number of DEGs and the percent change for each pathway in each animal model.

#### 2.6.3. Pathway Analysis of Association of Animal Models to Human Bulk RNA Sequencing and to Human scRNA Sequencing (CD4, CD8, B, NK)

Association analysis of animal models to human bulk RNA is applied with gene set enrichment analysis [25]. The gene set enrichment analysis leverages the signed ranks of gene changes (diseased vs. naïve group) from both animal models and human bulk RNA studies to assess whether the gene changes from animal models have the same or reverse direction of modulation to the gene changes from human bulk RNA. The association score for each pathway is reported based on a summation of the product of ranks at each gene common to both animal models and human bulk RNA study for a given pathway and is normalized to ensure the reported score bounded in the interval [−1, 1]. A normalized association score of −1 or +1 indicate all gene changes in animal models perfectly reverse or mimic the gene changes from human bulk RNA, respectively. Correspondingly, a value near 0 indicates a lack of connectivity between the genes change from animals to human bulk RNA. Gene expression data from publicly available GSE157293 were utilized to quantify DEGs in bulk RNA sequencing from renal samples of human LN patients [26].

Association analysis of animal models to human scRNA sequencing was applied with the same gene set enrichment analysis from above bulk RNA. The gene set enrichment analysis leverages the signed ranks of gene changes (diseased vs. naïve group) from both animal models and human scRNA sequencing study to assess whether the gene changes from animal models have the same or reverse direction of modulation than the gene changes from human scRNA sequencing in specific cell types (CD4, CD8, B, NK). Publicly available scRNA sequencing data were utilized from results published by the AMP-SLE Consortium (ImmPort SDY997) [5].

## 3. Results

### 3.1. Differential Gene Expression in Kidney Tissue from Animal Models of SLE/LN

**Figure 1:**
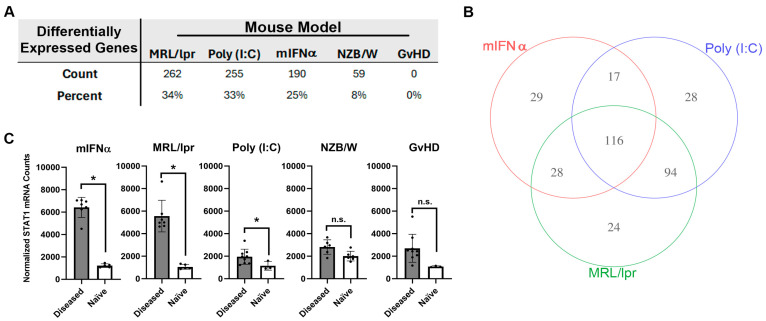
Analysis of autoimmunity- and inflammation-associated mRNA expression in kidney tissue harvested from animal models of SLE/LN. (**A**) DEGs in each animal model reported as both total count and percentage of all genes measured. (**B**) Venn diagram of significant genes identified in the mIFNα, poly (I:C) and MRL/lpr animal models of SLE/LN. Significance was determined using one-way ANOVA model at significance cutoff of adjusted *p*-value of 0.1 and at least 1.5-fold change between diseased and naïve mice. (**C**) Differential expression of *Stat1* mRNA between diseased and naïve mice for each animal model. Asterisks indicate significant differences. Bars indicate mean +/− standard deviation.

Differential expressions of 770 autoimmunity- and inflammation-associated genes were measured with the NanoString platform using kidney tissue harvested from five different mouse models of SLE/LN: MRL/lpr, poly (I:C), mIFNα, spontaneous NZB/W (referred to as NZB/W hereafter) and GvHD. Substantial variation in overall differential mRNA expression between diseased and naïve tissue was observed across these models. For the MRL/lpr, poly (I:C), mIFNα, NZB/W and GvHD models, 262, 255, 190, 59 and 0 differentially expressed genes (DEGs) were observed, respectively (Figure 1A and Figure S1). Some of the models produced gene signatures with significant overlap in differential expression. For example, 116 DEGs were shared between the MRL/lpr, poly (I:C) and mIFNα models (Figure 1B). However, some DEGs were unique to certain models, with the MRL/lpr, poly (I:C) and mIFNα having 24, 28 and 29 unique DEGs, respectively (Figure 1B and Table S1). Notably, the MRL/lpr, poly (I:C) and mIFNα all showed upregulation of *Stat1*, a commonly reported SLE-associated signal transducer and transcription factor downstream of Type I IFN signaling, while the NZB/W and GvHD models did not (Figure 1C) [27]. These findings indicate that, while significant similarities exist in the disease pathology of these animal models of SLE/LN, there are unique features of each model that should be understood by researchers wishing to use these models for drug discovery and autoimmune disease research.

### 3.2. Comparative Pathway Analysis between SLE/LN Animal Models

**Figure 2:**
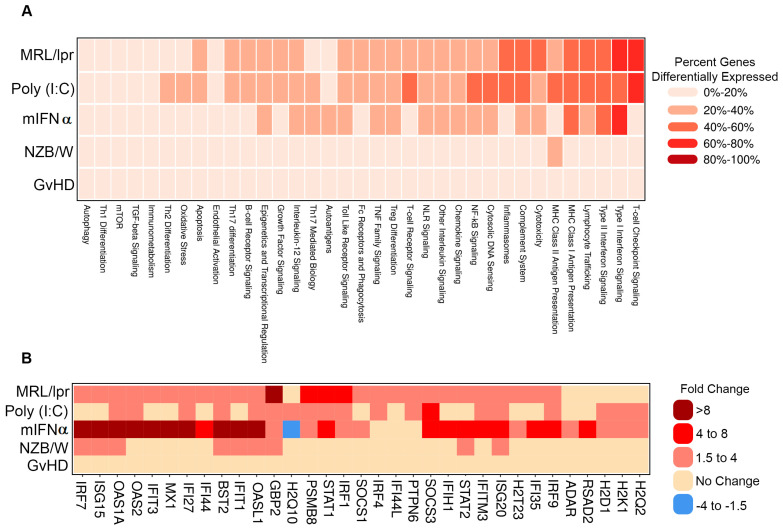
Pathway analysis of DEGs in animal models of SLE/LN. (**A**) Heatmap of modulated pathways (columns) for each animal model (rows). Colors binned by number of DEGs as a percentage of total genes for each pathway. (**B**) Heatmap of selected individual gene expression changes (columns) for genes in the Type I interferon signaling pathway, for each animal model (rows). Colors binned by fold changes (diseased vs. naïve mice) for each gene.

To further interrogate the similarities and differences between the gene signatures of each of the evaluated SLE/LN animal models, genes were analyzed as gene sets linked to autoimmune pathways. Several pathways showed concordant differential expression patterns across models, such as Type I interferon signaling, which was regulated in the MRL/lpr, poly (I:C) and mIFNα with 61% (27/44), 43% (19/44) and 68% (30/44) of genes in the pathway differentially expressed, respectively (Figure 2A). Notably, while this pathway was generally upregulated in each of these models, the amplitudes of change for key pathway genes *Oas1a* and *Mx1*, among others, were higher in the mIFNα (21.9- and 8.7-fold) model than the poly (I:C) (2.7- and 1.7-fold) or MRL/lpr (3.9- and 3.1-fold) models (Figure 2B). Additionally, modulation of some pathways was restricted to specific animal models. For example, expression of genes in the T cell checkpoint signaling pathway was substantially altered in the MRL/lpr (62% (32/52) of genes) and poly (I:C) (63% (33/52) of genes) models but not the mIFNα (17% (9/52) of genes) model. General T cell lineage markers *Cd3e* and *Cd3g* were upregulated in the MRL/lpr (7.3- and 7.8-fold) and poly (I:C) (3- and 4-fold) models, suggesting elevated T cell infiltrates in these models, a feature not observed in the mIFNα model (1.3- and 1.1-fold) (Figure S2A). Similarly, the MHC Class II Antigen Presentation pathway was heavily modulated in the MRL/lpr 38% (10/26) of genes) and poly (I:C) (42% (11/26) of genes) models but not in the mIFNα (12% (3/26) of genes) model (Figure 2A). Key MHC Class II Antigen Presentation pathway genes, *Cd74* and *Lag3*, which are associated with adaptive immune responses, were upregulated in the MRL/lpr (8.5- and 4.7-fold) and poly (I:C) (3.6- and 2.3-fold) models but not the mIFNα (1.1- and 1.2-fold) model (Figure S2B). These data support the conclusion that Type I interferon signaling, a key feature of SLE/LN in human disease, is captured by most of the animal models studied. However, substantial differences exist between the animal models with respect to the contribution of adaptive/innate immune cells to pathology in disease tissue. The suitability of these models of SLE/LN should be assessed based on the cell types involved and pathways under investigation, particularly in relation to specific drug discovery and translational research aims.

### 3.3. Association between Animal Tissue and Human Bulk Tissue or Single-Cell Gene Signatures in SLE Kidney

**Figure 3:**
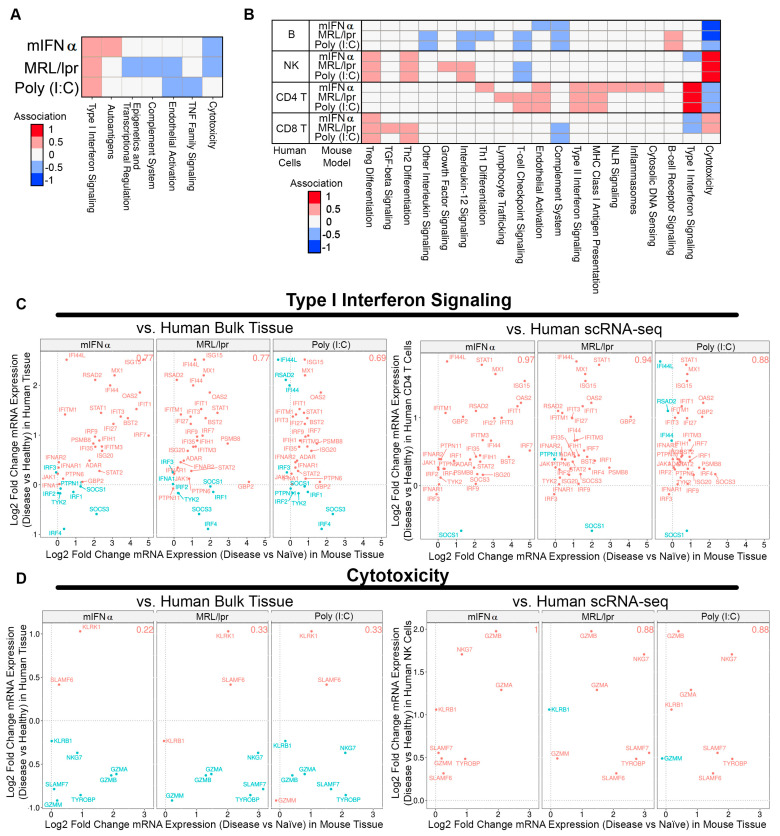
Comparing gene signatures of SLE/LN human bulk kidney tissue or scRNA-seq with SLE/LN animal model gene expression profiles. (**A**) Heatmap of associations between human bulk tissue and SLE/LN animal models (rows) for selected pathways (columns). (**B**) Heatmap of associations between human scRNA-seq cell types and SLE/LN animal models (rows) for selected pathways (columns). (**C**) Association plots for human bulk tissue (left, *y*-axis) or human CD4 T cells from scRNA-seq (right, *y*-axis) with animal model tissue (*x*-axis) for Type I interferon signaling pathway genes. Proportional association scores indicated for each comparison (top right). (**D**) Association plots for human bulk tissue (left, *y*-axis) or human NK cells from scRNA-seq (right, *y*-axis) with animal model tissue (*x*-axis) for cytotoxicity pathway genes. Proportional association scores indicated for each comparison (top right).

To further understand how well the animal models recapitulate SLE human disease features, DEGs from human SLE patient bulk kidney tissue and single-cell RNA sequencing (scRNA-seq) were associated with the gene expression profiles of each animal model (i.e., when human gene expression increases concordantly with animal gene expression, and vice versa) [5,28]. The MRL/lpr, poly (I:C), and mIFNα models exhibited significant DEGs compared to naïve controls, while the GvHD and spontaneous NZB/W models did not and were omitted from this analysis. When DEGs from human bulk kidney tissue were used, only moderate associations were observed, which were strongest for Type I interferon signaling (Figure 3A). However, when DEGs from human scRNA-seq were assessed, gene changes in subsets of human cell types showed markedly improved associations with gene changes in animal model tissue. For example, cytotoxicity pathway gene changes in SLE human NK cells showed a striking association with DEGs identified in kidney tissue from the MRL/lpr, poly (I:C) and mIFNα models. Similarly, changes in Type I interferon signaling pathway genes in SLE human CD4^+^ T cells showed strong associations with each animal model (Figure 3B). Notably, T cell-checkpoint-signaling DEGs in SLE human CD4^+^ T cells as well as B cell-receptor-signaling DEGs in SLE human B cells were positively associated with gene changes observed in the poly (I:C) and MRL/lpr models, but there was no association with gene changes in the mIFNα model (Figure 3B). Genes in the Type I interferon signaling pathway showed 0.77 (27/35) proportional association between the MRL/lpr model and human bulk kidney tissue, which increased to 0.94 (32/34) proportional association for human CD4^+^ T cells from scRNA-seq (Figure 3C). Similarly, genes in the cytotoxicity pathway showed 0.22 (2/9) proportional association between the mIFNα model and human bulk kidney tissue, which increased to 1.0 (8/8) proportional association for human NK cells from scRNA-seq (Figure 3D). These findings indicate that scRNA-seq may be more useful than bulk tissue gene expression changes for comparing specific disease pathways between species, especially when cell-type subsets are anticipated to contribute the majority of signaling for a given pathway in these models. Lastly, there were common pathways (NK cytotoxicity and CD4 IFN-I responses) amongst all three models compared to human disease while the MRL/lpr and poly (I:C) models engaged adaptive immune response pathways (B cell BRC and CD4^+^ T cell checkpoint signaling) in common with human SLE. These results may help provide a roadmap to identify appropriate SLE/LN mouse models to employ when targeting a specific pathway of interest.

## 4. Discussion

SLE is a complex, multiorgan system, autoimmune disease and drug development for it has been wrought with clinical trial failures for more than 40 years. While clinical trial failures in SLE can be attributed to heterogenous patient populations and high placebo response rates, there is also a component of pursuing targets where the mechanisms of action are not fully validated or aligned with the complexity of the disease state that has led to failures in the clinic. Some of the more recent failures in lupus trials include anti-CD20 (rituximab), anti-CD22 (epratuzumab), anti-BAFF (tabalumab, atacicept) CD28-Fc (abatacept), anti-IL6 (PF-04236921), and anti-IFN-α (sifalimumab) [29].

While mouse models of lupus have been invaluable for studying the disease and testing novel therapeutic agents, there is heterogeneity between the different models that make it difficult to select the correct model to test novel therapeutic targets. Here, we evaluated five common mouse models and compared their gene expression profiles against each other and to human lupus patient samples from the AMP-SLE database. The goal of this work is to help drug hunters or translational researchers identify the right SLE/LN model to run for their novel targets.

In this study, we used the NanoString platform to compare the differentially expressed genes in the kidneys between the spontaneous NZB/W, GvHD, MRL/lpr, poly (I:C), and mIFNα mouse models of SLE/LN. While there were 117 differentially expressed genes in common between the three models studied, 29 genes were unique for mIFNα, 28 unique genes for poly (I:C), and 24 unique genes for MRL/lpr (see Table S1 for full gene list).

DEG profiles from the mouse models tested here identified common and unique pathways altered in diseased mice compared to naïve controls. The Type I interferon signaling pathway was one such pathway identified as largely upregulated in the mIFN-α, poly (I:C), and MRL/lpr models (Figure 2A). Notably, the fold change for key pathway genes (e.g., *Oas1a* and *Mx1*) were expectedly highest in the mIFN-α model compared to poly (I:C) and MRL/lpr, which is consistent as these are IFN-induced genes (Figure 2B). Type I interferons have been reported to be elevated in both the poly (I:C) and MRL/lpr models [16,30]. However, modulation of T cell receptor, T cell checkpoint, and MHC II signaling pathways were restricted to MRL/lpr and poly (I:C), highlighting the contribution of the adaptive immune system to disease pathology in these models (Figure 2A, Figure S2). These results are consistent with published data which describe the nephritis in the mIFN-α model as predominantly myeloid cell infiltration in the renal interstitium [31], whereas MRL/lpr mice have a mixed population of myeloid cells and lymphocytes [32]. While Type I IFN signaling, a key feature of SLE/LN in human disease, is captured by most of these animal models, substantial differences exist between these models. The differential gene expression between these models emphasizes the need to evaluate their appropriateness for drug discovery and translational research based on cell types and pathways associated with human disease.

A low frequency of DEGs in NZB/W and absence of DEGs in GvHD models indicate low immune cell dysregulation in these models. The findings in the NZB/W model are in contrast to a comparative study between NZB/W and mIFN-α models that found relative abundance of renal inflammation in the NZB/W model consisting of activated leukocytes [31]. This discrepancy could be due to phenotypic drift, which has been reported in other SLE/LN models, or differences in gut microbiota/environmental factors between institutions [33,34]. Although the slow progression of the spontaneous NZB/W model may better resemble the decades-long progression of human SLE/LN, it can reduce their utility as a tool model for drug development. In the case of the spontaneous NZB/W model in our laboratory, onset and progression of disease varies widely among mice resulting in mild disease (Figure S3). To attain significant results, large investments in the number of mice and reagents would be required with considerable risk of failure after a prolonged study. Synchronizing the onset of disease and accelerating the progression of disease with mIFN-α or poly IC allow for these models to be more efficient but can skew the pathogenesis to different immune pathways and responses. Nevertheless, despite potential low immune cell involvement in NZB/W and GvHD models, they could still be valuable in understanding pathogenesis in LN patients with low-to-moderate interstitial inflammation or pauci-immune LN patients [4,35,36]. For this, using scRNA-seq for detailed analysis of non-immune cell DEGs would be more appropriate.

The primary goal of this work was to assess how well commonly used animal models recapitulate human SLE disease features. To this end, we compared how well differentially expressed genes from human SLE patient bulk kidney tissue and single-cell RNA sequencing (scRNA-seq) associated with mouse gene expression profiles in each animal model tested here. Using DEGs from human bulk RNA only identified Type I IFN signaling as strongly associated with the mIFNα, MRL/lpr, and poly (I:C) mouse models. Associations with these mouse models improved when compared to DEGs derived from scRNA-seq data from SLE patients. Two pathways stood out in all three models with strong correlation with human disease. Cytotoxicity pathway genes in NK cells and Type I IFN signaling genes in CD4^+^ T cells were highly associated with scRNA-seq-derived DEGs from SLE patients (Figure 3B). While the Type I IFN signaling genes were identified in both bulk and scRNA-seq analysis (Figure 3C), the cytotoxicity pathway was muted in the bulk analysis and did not show a positive association compared to the scRNA-seq data (Figure 3D). These findings suggest that scRNA-seq may provide more useful information when comparing specific disease pathways between human patient samples and mouse models, especially when cell-type subsets play a significant role.

While association with cytotoxicity in NK cells and CD4^+^ T cell IFN-I responses were common between the three models, the MRL/lpr and poly (I:C) stood out in two pathways. B cell receptor signaling genes in B cells and T cell checkpoint genes in CD4^+^ T cells were associated with human disease, thus further identifying the role of adaptive immune response in these models of SLE. These results reflect a proportional shift towards a mix population of innate and adaptive immune cells in the inflammatory response observed in the kidneys of the MRL/lpr and poly (I:C) models that is characteristic of human LN. The AMP SLE identified a mix population of macrophages, NK cells, T cells, and B cells in LN patient renal samples by scRNA-seq [5]. However, the AMP SLE study only evaluated a limited number of patient samples and may not sufficiently capture the diversity of immune response in human LN patients [5]. A study with a larger cohort of LN patients could capture rarer LN populations, such as the pauci-immune LN patients, allowing for deeper analysis into relationships between mouse models and human SLE/LN [35,36]. Lastly, it should be acknowledged that the microenvironment that these mice were exposed to could have an impact on the gene expression profiles we see here when compared to human patients not under strict quarantine.

## 5. Concluding Remarks

The goal of this work was to improve our understanding of mouse models of SLE/LN and how well they are associated with human disease. Using current technology in evaluating gene expression profiles from both mouse models and human disease, we hoped to improve our understanding of the molecular mechanisms involved in the pathogenesis of disease. The gene associations, emphasizing cell type-specific contributions in disease models, identified pathways common between mouse models and human disease. While this analysis is not exhaustive, nor does it cover all mouse models of SLE/LN [37], it helps to provide some insight into selecting appropriate mouse models of SLE/LN when targeting a specific pathway of interest for translational research and drug discovery. For example, if one were targeting a pathway that is present in CD4^+^ T cells, they may want to focus their preclinical studies on looking at Type I/II or T cell checkpoint signaling (Figure 3B). We hope others can use this as a guide to build better experiments to enrich their preclinical research and have improved translation guidance into clinical trials.

## Data Availability

Data are contained within the article or Supplementary Materials.

## Supplementary Materials

The following supporting information can be downloaded at https://www.mdpi.com/article/10.3390/biology13100778/s1. Table S1: DEG list of genes common to MRL/lpr, poly (I:C), and mIFNα; genes unique to MRL/lpr, poly (I:C), and mIFNα. Figure S1: Volcano plots of differential gene expression for each animal model of SLE compared to its non-disease control from Figure 1. Figure S2: Additional pathway analysis of differentially expressed genes in animal models of SLE/LN from Figure 2. Figure S3: ACR plots for individual NZB/W mice, highlighting variability of disease onset.
