# Supplementary material for: Translating Lupus: Comparative Transcriptional Profiles of Preclinical Lupus Models and Their Relevance to Human Disease
Source: Biology (Basel). 2024 Sep 28;13(10):778. doi: 10.3390/biology13100778 (PMC11505302; doi:10.3390/biology13100778)
Supplement: Supplementary file 1 [file biology-13-00778-s001.zip › biology-3148801-supplementary.pdf]

Supplementary Table S1. List of differentially expressed genes in common or unique to each animal model

Mouse Model						
Shared				MRL/lpr	Poly (I:C)	mIFNa
BCL2A1A	CXCL10	IL33	RIPK3	C9	AGER	ADAR
BST2	CXCR4	IRF1	SELE	CD163	AQP4	AGT
BTLA	CYBB	IRF9	SERPING1	CD19	BLK	C2
C1QA	DTX3L	ISG20	SIGLEC1	CD40LG	CD8A	C8G
C1QB	EBI3	ITGA4	SOCS3	CFD	CFH	CD24A
C1RA	EGR2	ITGAL	PTPRC	CXCR5	GUSB	CDH2
C3	EOMES	ITGAM	RELB	F8	H2DMB1	CLU
C4A	F13A1	ITGB2	RIPK3	FAS	HAVCR2	CTPS
C5AR1	FCGR1	KIF22	SELE	FASL	HDC	DLL4
C6	FCGR4	KLRK1	SERPING1	FCRL1	HIST1H2BK	EGF
C7	FCRLS	LIF	SIGLEC1	HIST1H2AO	IFNGR1	GRN
CASP1	FGR	LILRB4A	SOCS3	HIST1H4K	IL15	HIST2H3C2
CASP4	FPR1	LRR1	SPI1	IDO1	IL4RA	HSD11B1
CCL2	GBP2	MB21D1	STAT1	IFI44L	ITGA5	HSPB1
CCL3	GBP5	MMP3	STAT3	IL18R1	JUN	ID1
CCL4	GSDMD	MS4A4A	TAPBP	IL21	MAP3K14	IL10
CCL5	H2DMB2	NFKBIE	TCIRG1	IL2RA	MYD88	MSN
CCL7	H2M3	NKG7	TLR7	LTA	NFKBIA	NCAM1
CCR7	HIST1H3B	OAS1A	TLR9	NOD1	PADI2	NCR1
CD14	ICAM1	OAS2	TMEM173	PLAUR	PDPN	NR4A2
CD274	IFI27	OASL1	TNF	S100A9	PPARG	PTGS2
CD276	IFITM3	PANX1	TNFRSF1B	S1PR5	PYCARD	RSAD2
CD4	IKBKE	POU2F2	TNFSF13B	SELL	SDC1	SELENBP1
CD40	IKZF1	PRF1	TREM1	TRAT1	SDHA	SERPINA1A
CD68	IL10RA	PSMB10	TREX1		TGFBR2	TLR5
CD84	IL1RN	PSMB8	TRIM14		THBD	TMEM176A
CDKN1A	IKZF1	PRF1	TUBB5		WNT10B	TNFRSF10B
CFP	IL10RA	PSMB10	TYROBP		ZFP385A	TRIM25
CLEC4E	IL1RN	PSMB8	UBE2L6			TRIM35
CSF1	IL21R	PSMB9	UNC93B1			
CSF3R	IL2RB	PTPRC	VAV1			
CTSC	IL2RG	RELB	VCAM1			
			ZBP1			

There were 117 differentially expressed genes in common between MRL/lpr, poly (I:C), and mIFNα, 29 genes were unique for mIFNα, 28 unique genes for poly (I:C), and 24 unique genes for MRL/lpr.

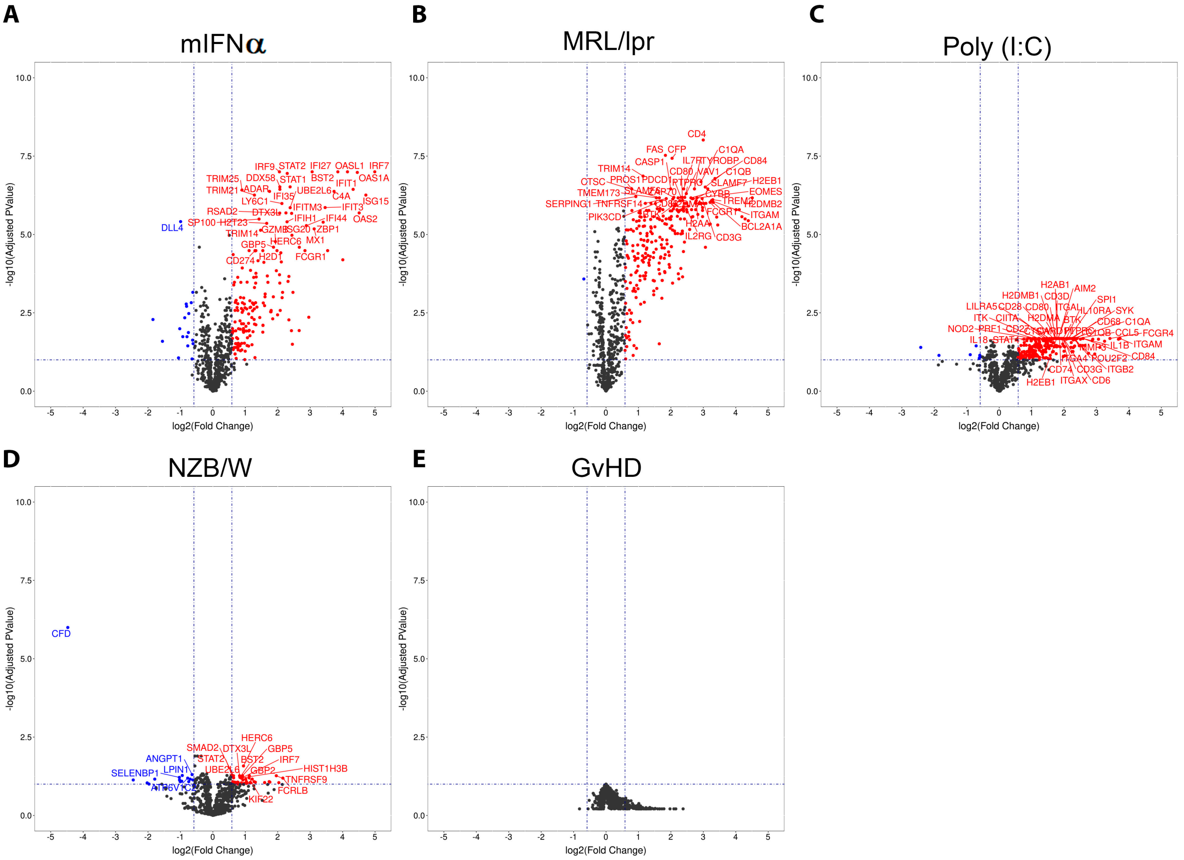

Supplementary Figure S1. Volcano plots of differential gene expression for each animal model of SLE compared to its non-disease control. (**A**) mIFNα, (**B**) MRL/lpr, (**C**) Poly (I:C), (**D**) NZB/W, (**E**) GvHD. Y-axis values are the -log10 transformed, multiplicity adjusted P values. X-axis values are log2 fold change.

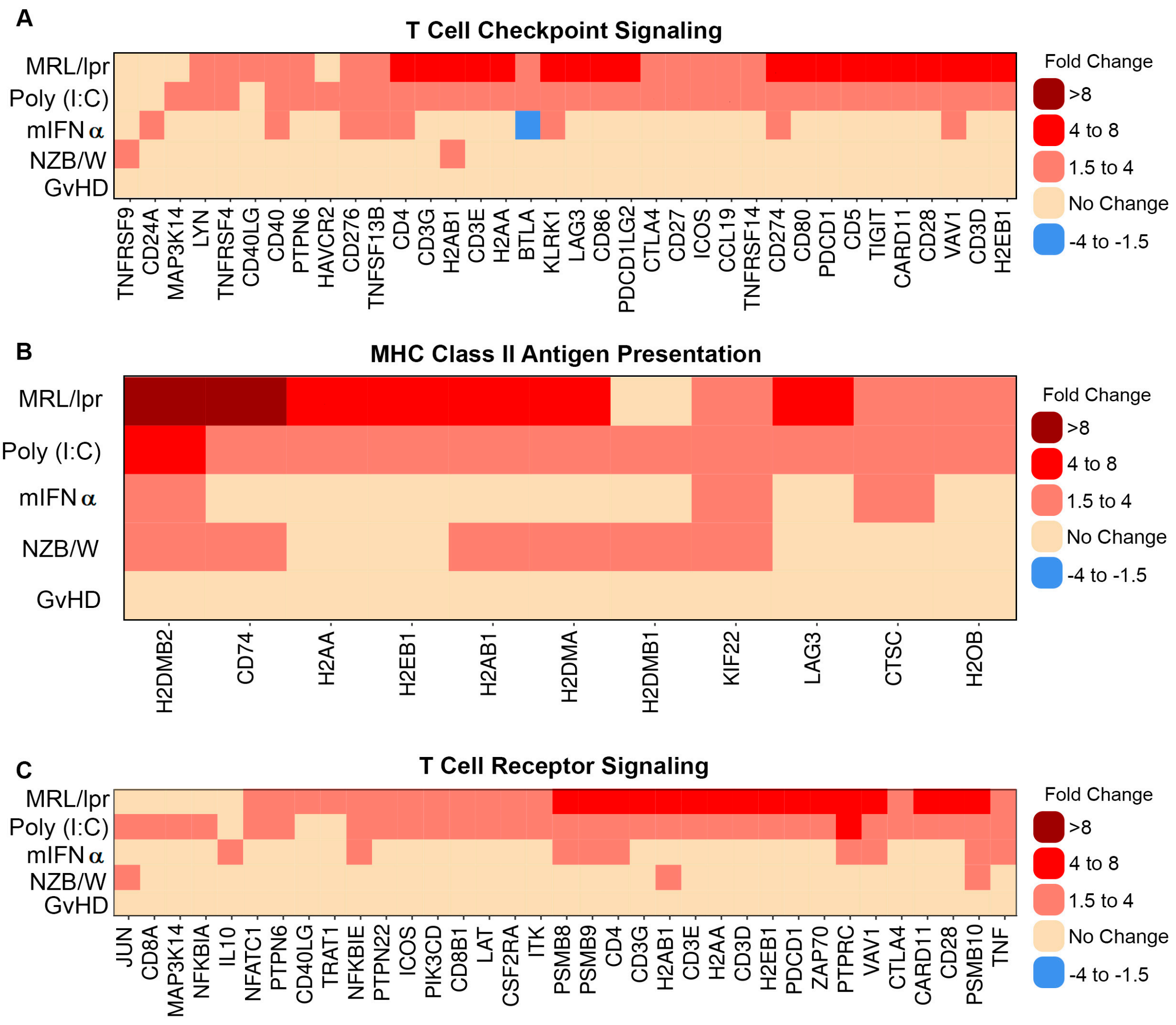

Supplementary Figure S2. Additional pathway analysis of differentially expressed genes in animal models of SLE/LN. (**A**) Heatmap of individual gene expression changes (columns) for selected genes in the T Cell Checkpoint Signaling pathway, for each animal model (rows). (**B**) Heatmap of individual gene expression changes (columns) for selected genes in the MHC Class II Antigen Presentation pathway, for each animal model (rows). Colors binned by fold changes (diseased vs naïve mice) for each gene. (**C**) Heatmap of individual gene expression changes (columns) for selected genes in the T Cell Receptor Signaling pathway, for each animal model (rows). Colors binned by fold changes (diseased vs naïve mice) for each gene.

A

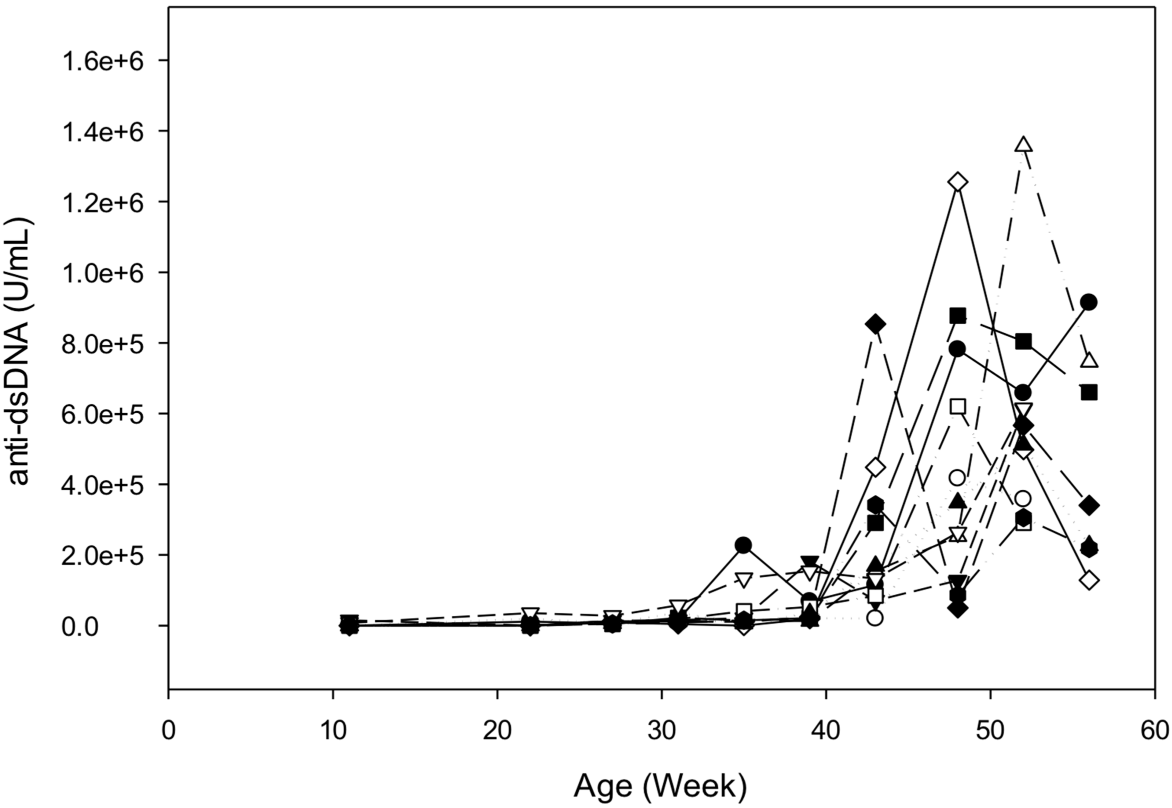

Supplementary Figure S3. Anti-double stranded DNA (anti-dsDNA) titers of individual female NZB/W mice with age. The development and rise of anti-dsDNA titers vary among mice signifying varied onset and progression of disease in the spontaneous NZB/W model of LN.
